# Supplementary material for: SARS-CoV-2 ferritin nanoparticle vaccines elicit broad SARS coronavirus immunogenicity
Source: bioRxiv. 2021 May 10:2021.05.09.443331. Preprint. [Version 1] doi: 10.1101/2021.05.09.443331 (PMC8132231; doi:10.1101/2021.05.09.443331)
Supplement: 1 [file NIHPP2021.05.09.443331V1-supplement-1.pdf]

bioRxiv preprint doi: https://doi.org/10.1101/2021.05.09.443331; this version posted May 10, 2021. The copyright holder for this preprint (which was not certified by peer review) is the author/funder. This article is a US Government work. It is not subject to copyright under 17 USC 105 and is also made available for use under a CC0 license.

List of supplementary figures.

Figure S1. Structure-based design of SARS-CoV-2 Spike-based ferritin nanoparticle immunogens and design pipeline, related to Figure 1.
Four ferritin nanoparticle immunogen designs were developed focused on (1) Spike ferritin nanoparticles (blue), (2) RBD ferritin nanoparticles (green), (3) RBD-NTD ferritin nanoparticles (black), and (4) S1 ferritin nanoparticles (orange). The design iterations and concepts are indicated, along with select mutations and design name. Lead vaccine candidates from each category are highlighted.

Figure S2. Negative-stain electron microscopy 2D micrographs of SARS-CoV-2 ferritin nanoparticle-based vaccine candidates, related to Figure 2 and 4.
Negative-stain electron microscopy 2D micrographs. The white scale bars represent 100 nm.
(A) Spike ferritin nanoparticles pCoV1B-05 and pCoV1B-08.
(B) RBD ferritin nanoparticles pCoV03, pCoV50, pCOV58, pCoV59, pCoV127, pCoV129, pCoV130, pCoV131
(C) RBD-NTD ferritin nanoparticles pCoV122, pCoV125, pCoV147
(D) S1 ferritin nanoparticle pCoV110 and pCoV112.

Figure S3. Biophysical and antigenic characterization of S-domain ferritin nanoparticle immunogens, related to Figure 2 and 3.
(A) Size-exclusion chromatography on a Superdex S200 10/300 column of representative SARS-CoV-2 Spike-based ferritin nanoparticles from the four design categories.
(B) Expression levels (mg/L supernatant) of representative SARS-CoV-2 Spike-based ferritin nanoparticles.
(C) Dynamic light scattering analysis of representative SARS-CoV-2 Spike-based ferritin nanoparticles.
(D) Spike ferritin nanoparticles (E) RBD ferritin, (F) RBD-NTD ferritin and (G) S1 ferritin nanoparticles were assessed for binding to a set of neutralizing antibodies (concentration = 30 μg/ml) by biolayer interferometry.

Figure S4 SARS-CoV-2 nanoparticle vaccine candidates elicit robust binding and pseudovirus neutralizing antibody responses in mice. Related to Figure 5 and 7.

bioRxiv preprint doi: https://doi.org/10.1101/2021.05.09.443331; this version posted May 10, 2021. The copyright holder for this preprint (which was not certified by peer review) is the author/funder. This article is a US Government work. It is not subject to copyright under 17 USC 105 and is also made available for use under a CC0 license.

(A) Biolayer Interferometry binding analysis of C57BL/6 and BALB/c sera from mice immunized with SpFN + Alhydrogel® (B) RFN + Alhydrogel® and (C) pCoV146 + Alhydrogel® to SARS-CoV-2 RBD. Mean values are indicated by a horizontal line, n=10.
(D) Pseudovirus neutralization (ID_{50} values) of C57BL/6 and BALB/c sera from mice immunized with SpFN + Alhydrogel® (E) RFN + Alhydrogel® and (F) pCoV146 + Alhydrogel®. Geometric mean values are indicated by a horizontal line, n=10.
(G) ELISA analysis of antibody isotype usage following immunization with SpFN + ALFQ (solid shapes), or SpFN + Alhydrogel® (open shapes). Sera collected study week 2, 5, and 8 from immunized mice were added in quadruplicate serial dilutions to ELISA plates coated with S-2P protein. Duplicated wells were probed with anti-mouse-IgG1-HRP. Additional duplicates were probed with either anti-mouse-IgG2c-HRP or anti-mouse IgG2a-HRP for C57BL/6 and BALB/c mice, respectively. Data was interpolated to obtain the dilution factor at OD_{450} of 1 and plotted as ratios of IgG2/IgG1. A horizontal dotted line denotes a balanced 1:1 IgG2/IgG1 ratio. Isotype ratio values were compared between the two adjuvant groups at each timepoint for each mouse type using a Mann-Whitney unpaired two-tailed non-parametric test.
(H) Biolayer interferometry analysis of BALB/c mouse sera binding to SARS-CoV-2 RBD at study weeks 2, 5 and 8. Mice were immunized with the four lead candidate vaccines SpFN (blue), RFN (green), pCoV146 (black) and pCOV111 (orange). Binding mean values are indicated by a horizontal line, n=10, sera responses at a given study week were compared for statistical differences using a Kruskal-Wallis test followed by a Dunn's post-test.
(I) ELISA analysis of BALB/c mice immune responses as indicated in (H). Binding geometric mean values of the endpoint titers are indicated by a horizontal line, n=10, sera responses at a given study week were compared for statistical differences using a Kruskal-Wallis test followed by a Dunn's post-test.
(J) Pseudovirus neutralization ID50 titers of BALB/c mice immunized as indicated in (H). Geometric mean values are indicated by a horizontal line, n=10, sera neutralization titers at a given study week for the four immunogens were compared for statistical differences using a Kruskal-Wallis test followed by a Dunn's post-test.
(K) Pseudovirus neutralization ID_{80} titers of C57BL/6 (left) and BALB/c mice (right) immunized as indicated in (H). Geometric mean values are indicated by a horizontal line, n=10, sera neutralization titers at a given study week for the four immunogens were compared for statistical differences using a Kruskal-Wallis test followed by a Dunn's post-test.
P values <0.0001 (****), <0.001 (***), <0.01 (**) or <0.05 (*).

Figure S5 SARS-CoV-2 SpFN vaccine candidate elicits robust binding and neutralizing antibody responses at reduced doses in mice. Related to Figure 5 and 7.
(A) Biolayer interferometry analysis of C57BL/6 and (B) BALB/c mouse sera binding response to SARS-CoV-2 RBD following immunization with reducing doses of SpFN.
(C, E) ELISA analysis of C57BL/6 and (D, F) BALB/c mouse sera binding response to SARS-CoV-2 RBD or S-2P following immunization with reducing doses of SpFN.
(G) SARS-CoV-2 pseudovirus ID80 neutralization titers of mice immunized with 0.08 µg SpFN + ALFQ.
(H) Authentic SARS-CoV-2 virus ID80 neutralization titers of mice immunized with 10 µg (blue) or 0.08 µg (light blue) SpFN + ALFQ. Geometric mean titers for each group and time

bioRxiv preprint doi: https://doi.org/10.1101/2021.05.09.443331; this version posted May 10, 2021. The copyright holder for this preprint (which was not certified by peer review) is the author/funder. This article is a US Government work. It is not subject to copyright under 17 USC 105 and is also made available for use under a CC0 license.

point are indicated by a horizontal line, n =10. Neutralization titers for the two dose groups at each study time point were compared for statistically significant differences using a Mann-Whitney unpaired two-tailed non-parametric test. The two BALB/c time points that showed differences are indicated by bars. P values <0.001 (***), <0.01 (**). (I) Mouse sera from study week 10 was analyzed for hACE2 blocking capacity to SARS-CoV-2 RBD (left) or SARS-CoV-1 RBD using a biolayer interferometry assay format.

Figure S6 Histopathological analysis of SARS-CoV-2 infection in K18-ACE2 mice. Related to Figure 7

(A, B) Hematoxylin and eosin staining of lung sections from K18-hACE2 mice following intranasal infection with 1.25 x 10^4 PFU SARS-CoV-2. Images show two magnifications. Images are representative of n = 10 per group.

bioRxiv preprint doi: https://doi.org/10.1101/2021.05.09.443331; this version posted May 10, 2021. The copyright holder for this preprint (which was not certified by peer review) is the author/funder. This article is a US Government work. It is not subject to copyright under 17 USC 105 and is also made available for use under a CC0 license.

Table S1. Spike-domain ferritin immunogens

Spike-Ferritin (all based on S-2P variant with Δfurin and PP)

Construct ID	Description		
pCoV1B-01	S2P(1-1137)-del-4-Ferritin	Shortened ectodomain - no coiled coil (closest to flu HA pass off)	NL
pCoV1B-02	S2P(1-1137)-del-6-Ferritin	Shortened ectodomain - no coiled coil (closest to flu HA pass off)	NL
pCoV1B-03	S2P(1-1208)-del-Ferritin	Full ectodomain	NL
pCoV1B-04	S2P(1-1208)-GCN4-Ferritin	Full ectodomain with GCN4	NL
pCoV1B-05	S2P(1-1154)-del-Ferritin	Shortened ectodomain with ending with a couple turns of coiled coil	NL
pCoV1B-06	S2P(1-1158)op1-del-Ferritin	Optimized HR ending (end on glycan N1158)	NL
pCoV1B-07	S2P(1-1158)op2-del-Ferritin	Optimized HR ending (Ile) (end on glycan N1158)	NL
pCoV1B-08	S2P(1-1158)op1x2-del-Ferritin	Optimized HR ending (N1158 glycan removed, but exists on the repeated HR)	NL
pCoV1B-09	S2P(1-1158)op2x2-del-Ferritin	Optimized HR ending (Ile) (N1158 glycan removed, but exists on the repeated HR)	NL
pCoV1B-10	S2P(1-1158)op1-fGCN4-del-Ferritin	Optimized HR ending with GCN4 fused in register (no glycan N1158)	NL
pCoV1B-01-PL	PL-S2P(12-1137)-del-4-Ferritin	Shortened ectodomain - no coiled coil (closest to flu HA pass off)	PL
pCoV1B-02-PL	PL-S2P(12-1137)-del-6-Ferritin	Shortened ectodomain - no coiled coil (closest to flu HA pass off)	PL
pCoV1B-03-PL	PL-S2P(12-1208)-del-Ferritin	Full ectodomain	PL
pCoV1B-04-PL	PL-S2P(12-1208)-GCN4-Ferritin	Full ectodomain with GCN4	PL
pCoV1B-05-PL	PL-S2P(12-1154)-del-Ferritin	Shortened ectodomain with ending with a couple turns of coiled coil	PL
pCoV-1B-06-PL (aka SpFN)	PL-S2P(12-1158)op1-del-Ferritin	Optimized HR ending (end on glycan N1158)	PL
pCoV1B-07-PL	PL-S2P(12-1158)op2-del-Ferritin	Optimized HR ending (Ile) (end on glycan N1158)	PL
pCoV1B-08-PL	PL-S2P(12-1158)op1x2-del-Ferritin	Optimized HR ending (N1158 glycan removed, but exists on the repeated HR)	PL
pCoV1B-09-PL	PL-S2P(12-1158)op2x2-del-Ferritin	Optimized HR ending (Ile) (N1158 glycan removed, but exists on the repeated HR)	PL
pCoV1B-10-PL	PL-S2P(12-1158)op1-fGCN4-del-Ferritin	Optimized HR ending with GCN4 fused in register (no glycan N1158)	PL

RBD-Ferritin

Construct ID	Description	Comment	Leader
pCoV03	His8-3C-RBD(331-527)-Ferritin	N-terminal His8 with HRV-3C cleavage site, GSGGGG linker between RBD and Ferritin	PL
pCoV29	His8-3C-RBD-3-Ferritin	SGG linker	PL
pCoV30	His8-3C-RBD-3-del-Ferritin	SGG linker, Δ first 10 residues in ferritin, then DIEK changed to DIIK	PL
pCoV31	His8-3C-RBD-6-del-Ferritin	P527G, Δ first 8 residues in ferritin, then SKDIEK changed to DIIK	PL
pCoV1A-01	His8-3C-RBD-PPII-Ferritin	Extend distance between RBD and ferritin - using polyproline Helix	PL
pCoV1A-02	His8-3C-RBD-alpha1-Ferritin	Extend distance between RBD and ferritin - using alpha Helix from bottom of S protein	PL
pCoV1A-03	His8-3C-RBD-alpha2-Ferritin	Extend distance between RBD and ferritin- using alpha Helix from bottom of S protein	PL
pCoV1A-04	His8-3C-RBD-GCN4-del-Ferritin	Extend distance between RBD and ferritin + stabilize ferritin - using GCN4 trimerization motif	PL

bioRxiv preprint doi: https://doi.org/10.1101/2021.05.09.443331; this version posted May 10, 2021. The copyright holder for this preprint (which was not certified by peer review) is the author/funder. This article is a US Government work. It is not subject to copyright under 17 USC 105 and is also made available for use under a CC0 license.

pCoV1A-05	His8-3C-RBD-1141_1158op1-del-Ferritin	Extend distance between RBD and ferritin + stabilize ferritin - using semi-native trimerization motif	PL
pCoV1A-06	His8-3C-RBD-1141_1158op1x2-del-Ferritin	Extend distance between RBD and ferritin + stabilize ferritin - using semi-native trimerization motif	PL
pCoV49	His8-3C-RBD-F456N/K458T-Ferritin	RBD with indicated point mutations	PL
pCoV50	His8-3C-RBD-L455R/Y449K/F490R-Ferritin	RBD with indicated point mutations	PL
pCoV51	His8-3C-RBD-L455R-Ferritin	RBD with indicated point mutation	PL
pCoV52	His8-3C-RBD-I468R-Ferritin	RBD with indicated point mutation	PL
pCoV53	His8-3C-RBD-Y453R-Ferritin	RBD with indicated point mutation	PL
pCoV54	His8-3C-RBD-L452R-Ferritin	RBD with indicated point mutation	PL
pCoV55	His8-3C-RBD-L492R-Ferritin	RBD with indicated point mutation	PL
pCoV56	His8-3C-RBD-F490R-Ferritin	RBD with indicated point mutation	PL
pCoV57	His8-3C-RBD-F490A-Ferritin	RBD with indicated point mutation	PL
pCoV58	His8-3C-RBD-L518N/L519K/H520S-Ferritin	RBD with indicated point mutations	PL
pCoV59	His8-3C-RBD-L518R-Ferritin	RBD with indicated point mutation	PL
pCoV60	His8-3C-RBD-V367T/L335N-Ferritin	RBD with indicated point mutations	PL
pCoV61	His8-3C-RBD-T385N/L387T-Ferritin	RBD with indicated point mutations	PL
pCoV62	His8-3C-RBD-V382R-Ferritin	RBD with indicated point mutation	PL
pCoV63	His8-3C-RBD-F377R-Ferritin	RBD with indicated point mutation	PL
pCoV127	His8-3C-RBD-F490A/L518N/L519K/H520S-Ferritin	RBD with indicated point mutations	PL
pCoV128	His8-3C-RBD-F490A/L518R-Ferritin	RBD with indicated point mutations	PL
pCoV129	His8-3C-RBD-L455R/Y449K/F490R/L518N/L519K/H520S-Ferritin	RBD with indicated point mutations	PL
pCoV130	His8-3C-RBD-L455R/Y449K/F490R/L518R-Ferritin	RBD with indicated point mutations	PL
pCoV131 (aka RFN)	His8-3C-RBD-Y453R/L518N/L519K/H520S-Ferritin	RBD with indicated point mutations	PL
pCoV132	His8-3C-RBD-Y453R/L518R-Ferritin	RBD with indicated point mutations	PL

RBD-NTD-Ferritin

Construct ID	Description		
pCoV122	His8-3C-RBD(331-527)-GSGGSG-NTD(12-303)-Ferritin	N-terminal HIs8 with HRV-3C cleavage site, GSGGSG linker between RBD and NTD, GSGGGG linker between NTD and Ferritin	PL
pCoV123	His8-3C-RBD-F490R-NTD-Ferritin	RBD with indicated point mutation	PL
pCoV124	His8-3C-RBD-F490A-NTD-Ferritin	RBD with indicated point mutation	PL
pCoV125	His8-3C-RBD-L518N/L519K/H520S-NTD-Ferritin	RBD with indicated point mutations	PL
pCoV126	His8-3C-RBD-L518R-NTD-Ferritin	RBD with indicated point mutation	PL
pCoV146	His8-3C-RBD-Y453R-L518N/L519K/H520S-NTD-Ferrritin	RBD with indicated point mutations	PL
pCoV147	His8-3C-RBD-F490A-L518N/L519K/H520S-NTD-Ferrritin	RBD with indicated point mutations	PL

bioRxiv preprint doi: https://doi.org/10.1101/2021.05.09.443331; this version posted May 10, 2021. The copyright holder for this preprint (which was not certified by peer review) is the author/funder. This article is a US Government work. It is not subject to copyright under 17 USC 105 and is also made available for use under a CC0 license.

S1-Ferritin

Construct ID	Description		
pCoV68	S1(12-678)-Ferritin	GSGGSG linker between S1 and Ferritin	PL
pCoV107	S1(12-655)-Ferritin	24 residues removed from the C-terminus	PL
pCoV108	S1(12-655)-L611N/Q613T-Ferritin	24 residues removed from the C-terminus, S1 with indicated point mutations	PL
pCoV109	S1(12-696)-Ferritin	Extended the sequence to include a portion of S2	PL
pCoV110	S1(12-676)-G-S2(689-696)-Ferritin	Extended the sequence to include a portion of S2 with the indicated leader between the two regions	PL
pCoV111	S1(12-676)-GG-S2(689-696)-Ferritin	Extended the sequence to include a portion of S2 with the indicated leader between the two regions	PL
pCoV112	S1(12-676)-PG-S2(689-696)-Ferritin	Extended the sequence to include a portion of S2 with the indicated leader between the two regions	PL
pCoV113	S1-Y312N/Q313Y/T314T-Ferritin	S1 with indicated point mutations	PL
pCoV114	S1-I651N/A653S-Ferritin	S1 with indicated point mutations	PL
pCoV115	S1-S316C/V595C-Ferritin	S1 with indicated point mutations	PL
pCoV116	S1-V320C/S591C-Ferritin	S1 with indicated point mutations	PL
pCoV117	S1-L560Q/F562H-Ferritin	S1 with indicated point mutations	PL
pCoV118	S1-F562N/Q564T-Ferritin	S1 with indicated point mutations	PL
pCoV119	S1-F490R-Ferritin	S1 with indicated point mutation	PL
pCoV120	S1-F490A-Ferritin	S1 with indicated point mutation	PL
pCoV02	S1(16-678)-Ferritin	4 residues removed from N-terminus	PL
pCoV67	His8-3C-S1-Ferritin	His8 and HRV-3C cleavage site added to N-terminus	PL

bioRxiv preprint doi: https://doi.org/10.1101/2021.05.09.443331; this version posted May 10, 2021. The copyright holder for this preprint (which was not certified by peer review) is the author/funder. This article is a US Government work. It is not subject to copyright under 17 USC 105 and is also made available for use under a CC0 license.

Table S2. Negative-stain Electron Microscopy Data Collection and Refinement

Protein	SpFN_1B-06-PL	RFN_131	pCoV146	pCoV111	pCoV1B-05
Immunogen Fused	Spike (S2P)	RBD	RBD-NTD	S1	Spike (S2P)
EMPIAR Code	XXXXX	XXXXX	XXXXX	XXXXX	XXXXX
EMDB Code	XXXX	XXXX	XXXX	XXXX	XXXX
**Data Collection**					
Microscope	Tecnai T20	Tecnai T20	Tecnai T20	Tecnai T20	Talos L120C
Voltage (kV)	200 kV	200 kV	200 kV	200 kV	120 kV
Camera	Eagle 4K	Eagle 4K	Eagle 4K	Eagle 4K	Ceta
Software	SerialEM	SerialEM	SerialEM	SerialEM	EPU
Pixel Size (Å/pix)	2.195	2.195	2.195	2.195	2.542
Underfocus range	0.7-1.3	0.8-1.3	0.6-1.5	0.8-1.6	0.5-0.9
**Image Processing**					
Software	RELION 3.0.8	RELION 3.0.8	RELION 3.0.8	RELION 3.0.8	RELION 3.1.1
# Particle Images	11502	3383	832	2121	2143
Pixel Size (Å/pixel)	4.39	4.39	4.39	4.39	5.084
Box Size (pixels)	160	160	160	160	200
Symmetry (3D)	O	O	O	O	--
Initial Lowpass (Å) (RELION)	100	80	100	100	--
High-res Limit (Å) (cisTEM)	--	--	--	--	--
Resolution (Å)	25	21	30	30	--

bioRxiv preprint doi: https://doi.org/10.1101/2021.05.09.443331; this version posted May 10, 2021. The copyright holder for this preprint (which was not certified by peer review) is the author/funder. This article is a US Government work. It is not subject to copyright under 17 USC 105 and is also made available for use under a CC0 license.

Table S3. Mouse immunogenicity study immunogens, adjuvants, and mouse type

pCOV no.	Immunogen design category, Study design	C57BL/6 ALFQ	Balb/c ALFQ	C57BL/6 Alhydrogel	Balb/c Alhydrogel
1B-05	S-Trimer-Ferritin	X	X		
1B-06-PL	S-Trimer-Ferritin	X	X	X	X
**RBD-Ferritin constructs**					
pCOV no.	Immunogen design category, Study design	C57BL/6 ALFQ	Balb/c ALFQ	C57BL/6 Alhydrogel	Balb/c Alhydrogel
50	RBD-Ferritin		X		
58	RBD-Ferritin	X	X	X	X
59	RBD-Ferritin		X		
127	RBD(57+58)-Ferritin	X	X	X	X
129	RBD(50+58)-Ferritin	X	X	X	X
130	RBD(50+59)-Ferritin		X		
131	RBD(53+58)-Ferritin	X	X	X	X
**S1-Ferritin constructs**					
pCOV no.		C57BL/6 ALFQ	Balb/c ALFQ	C57BL/6 Alhydrogel	Balb/c Alhydrogel
111	S1-Ferritin	X	X		
**RBD-NTD-Ferritin constructs**					
pCOV no.	Immunogen design category, Study design	C57BL/6 ALFQ	Balb/c ALFQ	C57BL/6 Alhydrogel	Balb/c Alhydrogel
122	RBD-NTD-Ferritin	X	X		
125	RBD(58)-NTD-Ferritin		X		X
146	RBD(53+58)-NTD-Ferritin	X	X	X	X
147	RBD(57+58)-NTD-Ferritin	X			

bioRxiv preprint doi: https://doi.org/10.1101/2021.05.09.443331; this version posted May 10, 2021. The copyright holder for this preprint (which was not certified by peer review) is the author/funder. This article is a US Government work. It is not subject to copyright under 17 USC 105 and is also made available for use under a CC0 license.

Table S4. Animal immunogenicity SARS-CoV-2 pseudovirus neutralization ID50 and ID80
Numbers shown are the ID50/ID80 geometric mean titers for a group, with study week 2, 5, and 8 shown in vertical order.

pCOV no.	Immunogen design category, Study design	C57BL/6 ALFQ	Balb/c ALFQ	C57BL/6 Alhydrogel	Balb/c Alhydrogel
1B-05	S-Trimer-Ferritin (x 2 groups)	702/189<br>8,709/2,346<br>13,076/5,647	115/<80<br>3,934/716<br>5,546/1,447		
1B-06-PL	S-Trimer-Ferritin	14,976/5,396<br>41,237/16,8184<br>7,323/16,52	1,152/355<br>16,816/6,662<br>25,062/6,540		
**RBD-Ferritin constructs**					
pCOV no.	Immunogen design category, Study design	C57BL/6 ALFQ	Balb/c ALFQ	C57BL/6 Alhydrogel	Balb/c Alhydrogel
50	RBD-Ferritin		X		
58	RBD-Ferritin	577/238<br>11,224/2,793<br>31,562/10,09	353/123<br>13,466/3,802<br>25,340/7,692	293/211<br>1,734/688<br>5,097/1261	232/<80<br>4,836/1,086<br>9,439/2,569
59	RBD-Ferritin		X		
127	RBD(57+58)-Ferritin	X	X	X	X
129	RBD(50+58)-Ferritin	X	X	X	X
130	RBD(50+59)-Ferritin		X		
131	RBD(53+58)-Ferritin	358/107<br>15,950/5,667<br>38,110/12,824	270/95<br>13,090/3,539<br>32,969/10,079	682/163<br>1,181/403<br>2,845/529	119/<40<br>182/103<br>240/99
**S1-Ferritin constructs**					
pCOV no.		C57BL/6 ALFQ	Balb/c ALFQ	C57BL/6 Alhydrogel	Balb/c Alhydrogel
111	S1-Ferritin	1,770/350<br>14,893/3,636<br>19,157/5,564	450/172<br>18,112/3,846<br>17,108/3,886		
**RBD-NTD-Ferritin constructs**					
pCOV no.	Immunogen design category, Study design	C57BL/6 ALFQ	Balb/c ALFQ	C57BL/6 Alhydrogel	Balb/c Alhydrogel
122	RBD-NTD-Ferritin	X	X		
125	RBD(58)-NTD-Ferritin		X		X
146	RBD(53+58)-NTD-Ferritin	230/91<br>16,678/4,356<br>20,107/6,126	240/89<br>31,252/7,190<br>24,854/6,744	<80/<80<br>667/460<br>940/289	662/<80<br>2,087/537<br>2,417/701
147	RBD(57+58)-NTD-Ferritin	X			

bioRxiv preprint doi: https://doi.org/10.1101/2021.05.09.443331; this version posted May 10, 2021. The copyright holder for this preprint (which was not certified by peer review) is the author/funder. This article is a US Government work. It is not subject to copyright under 17 USC 105 and is also made available for use under a CC0 license.

Figure S1

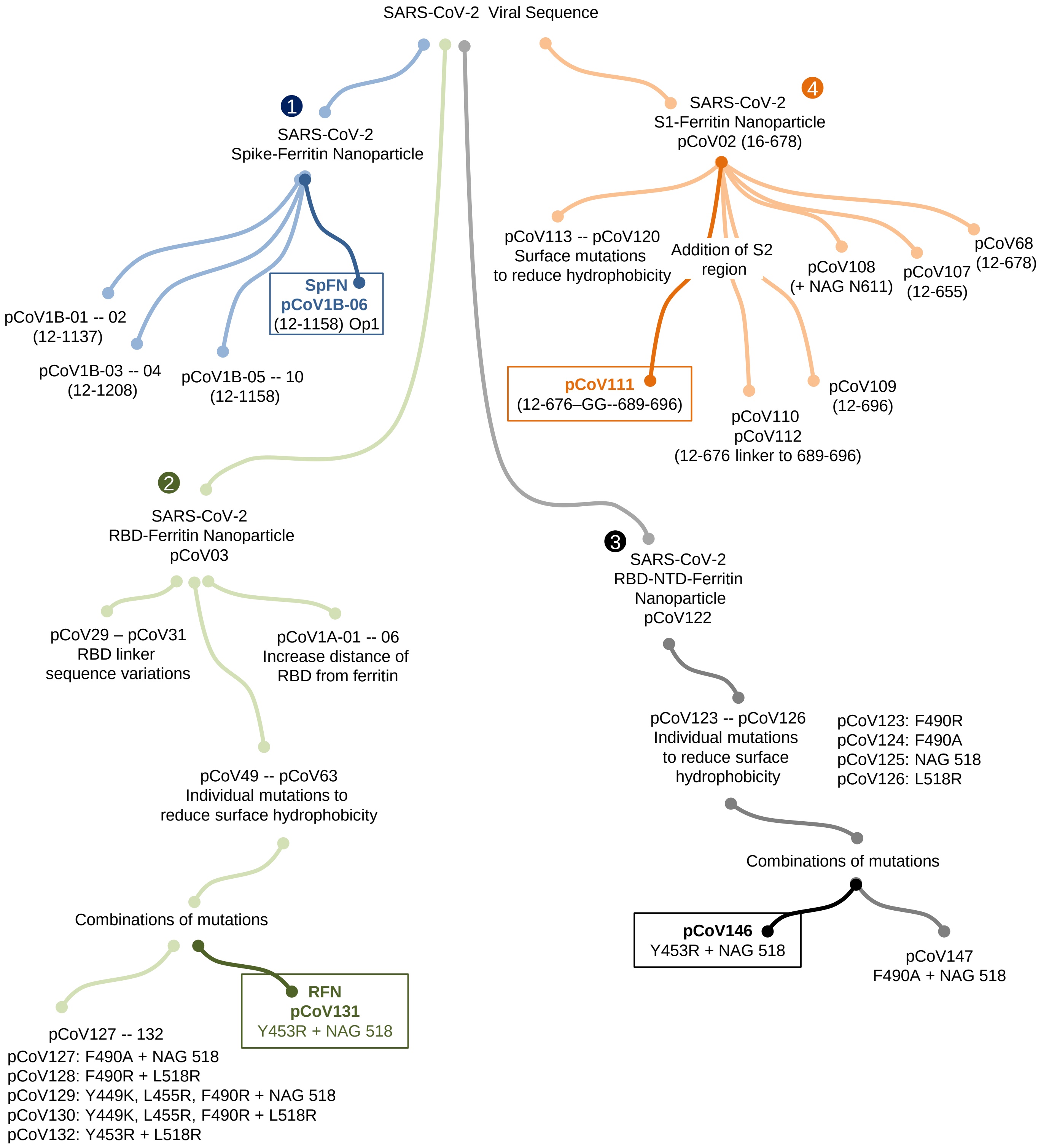

Figure S1. Structure-based design of SARS-CoV-2 S-domain ferritin nanoparticle immunogens and design pipeline. Related to Figure 1.
Four ferritin nanoparticle immunogen designs were developed focused on (1) Spike ferritin nanoparticles (blue), (2) RBD ferritin nanoparticles (green), (3) RBD-NTD ferritin nanoparticles (black), and (4) S1 ferritin nanoparticles (orange). The design iterations and concepts are indicated, along with select mutations and design name. Lead vaccine candidates from each category are highlighted.

bioRxiv preprint doi: https://doi.org/10.1101/2021.05.09.443331; this version posted May 10, 2021. The copyright holder for this preprint (which was not certified by peer review) is the author/funder. This article is a US Government work. It is not subject to copyright under 17 USC 105 and is also made available for use under a CC0 license.

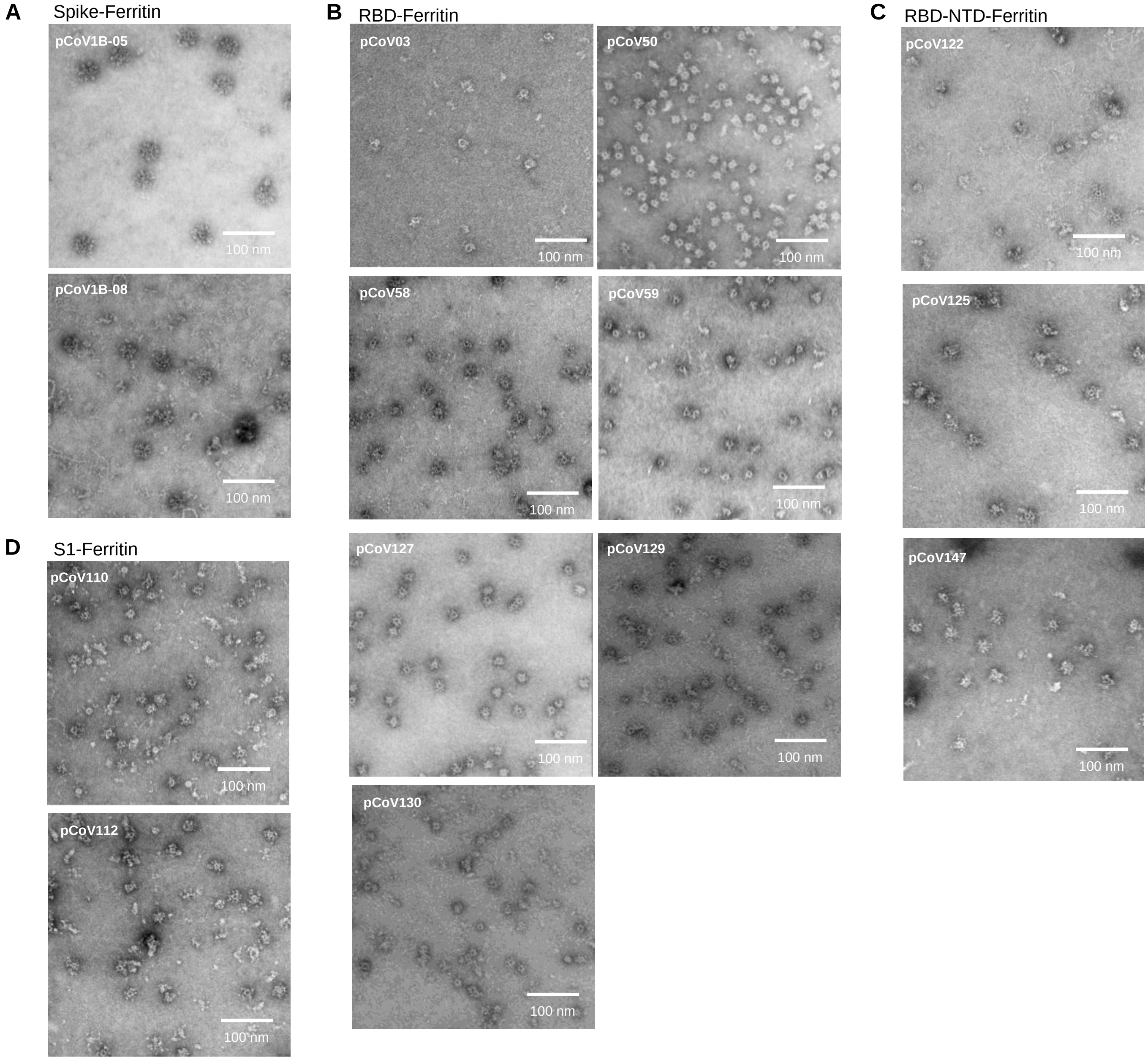

Figure S2. Negative-stain electron microscopy 2D micrographs of SARS-CoV-2 ferritin nanoparticle vaccine candidates, related to Figure 2 and 4.
Negative-stain electron microscopy 2D micrographs. The white scale bars represent 100 nm.
(A) Spike ferritin nanoparticles pCoV1B-05 and pCoV1B-08.
(B) RBD ferritin nanoparticles pCoV03, pCoV50, pCOV58, pCoV59, pCoV127, pCoV129, pCoV130, pCoV131
(C) RBD-NTD ferritin nanoparticles pCoV122, pCoV125, pCoV147
(D) S1 ferritin nanoparticle pCoV110 and pCoV112.

bioRxiv preprint doi: https://doi.org/10.1101/2021.05.09.443331; this version posted May 10, 2021. The copyright holder for this preprint (which was not certified by peer review) is the author/funder. This article is a US Government work. It is not subject to copyright under 17 USC 105 and is also made available for use under a CC0 license.

Figure S3

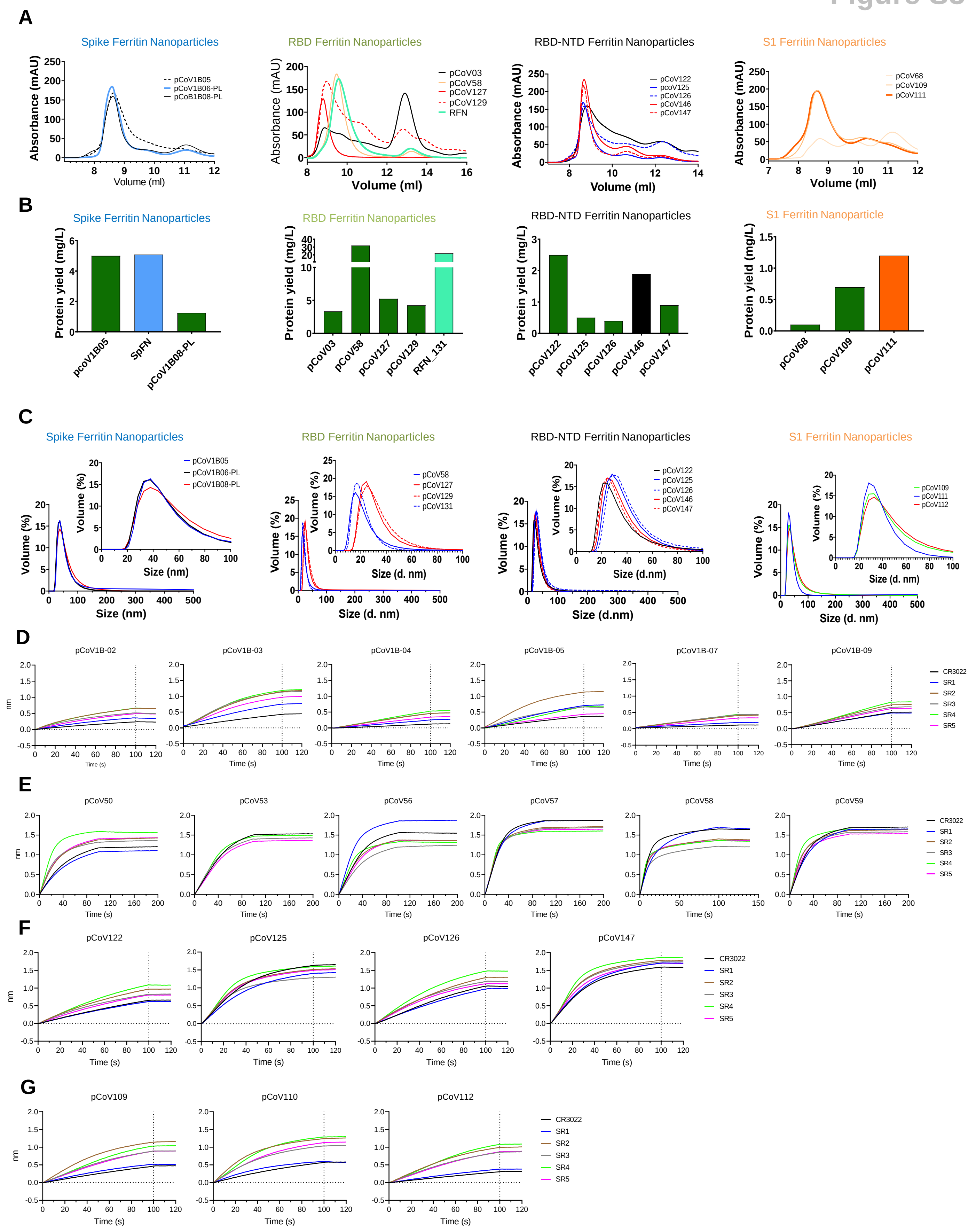

Figure S3. Biophysical and antigenic characterization of S-domain ferritin nanoparticle immunogens. Related to Figure 2 and 3.

(A) Size-exclusion chromatography on a Superdex S200 10/300 column of representative SARS-CoV-2 S-based ferritin nanoparticles from the four design categories.

(B) Expression levels (mg/L supernatant) of representative SARS-CoV-2 Spike-based ferritin nanoparticles.

(C) Dynamic light scattering analysis of representative SARS-CoV-2 Spike-based ferritin nanoparticles.

(D) Spike ferritin nanoparticles (E) RBD ferritin, (F) RBD-NTD ferritin and (G) S1 ferritin nanoparticles were assessed for binding to a set of neutralizing antibodies (concentration = 30 µg/ml) by biolayer interferometry.

bioRxiv preprint doi: https://doi.org/10.1101/2021.05.09.443331; this version posted May 10, 2021. The copyright holder for this preprint (which was not certified by peer review) is the author/funder. This article is a US Government work. It is not subject to copyright under 17 USC 105 and is also made available for use under a CC0 license.

Figure S4

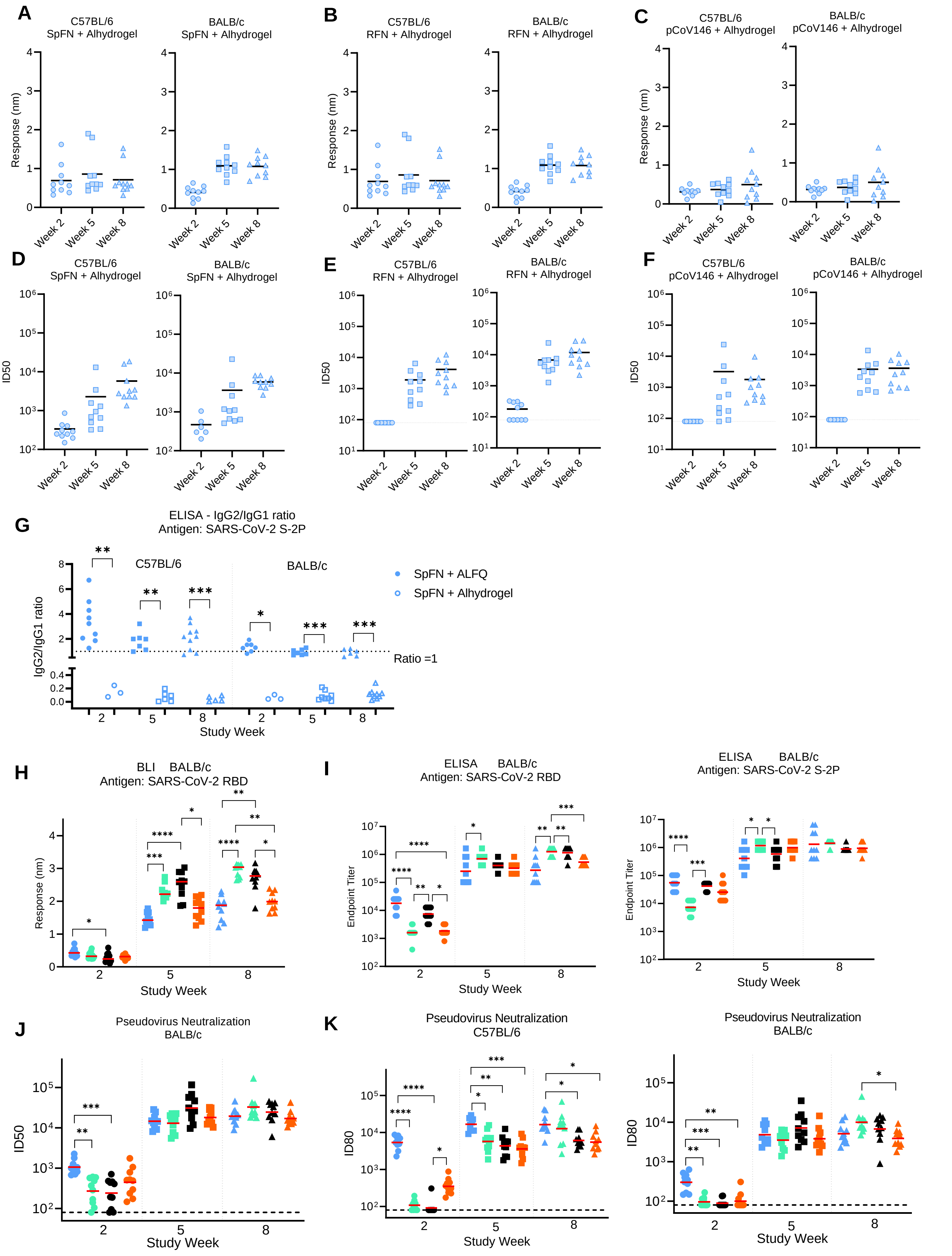

bioRxiv preprint doi: https://doi.org/10.1101/2021.05.09.443331; this version posted May 10, 2021. The copyright holder for this preprint (which was not certified by peer review) is the author/funder. This article is a US Government work. It is not subject to copyright under 17 USC 105 and is also made available for use under a CC0 license.

Figure S5

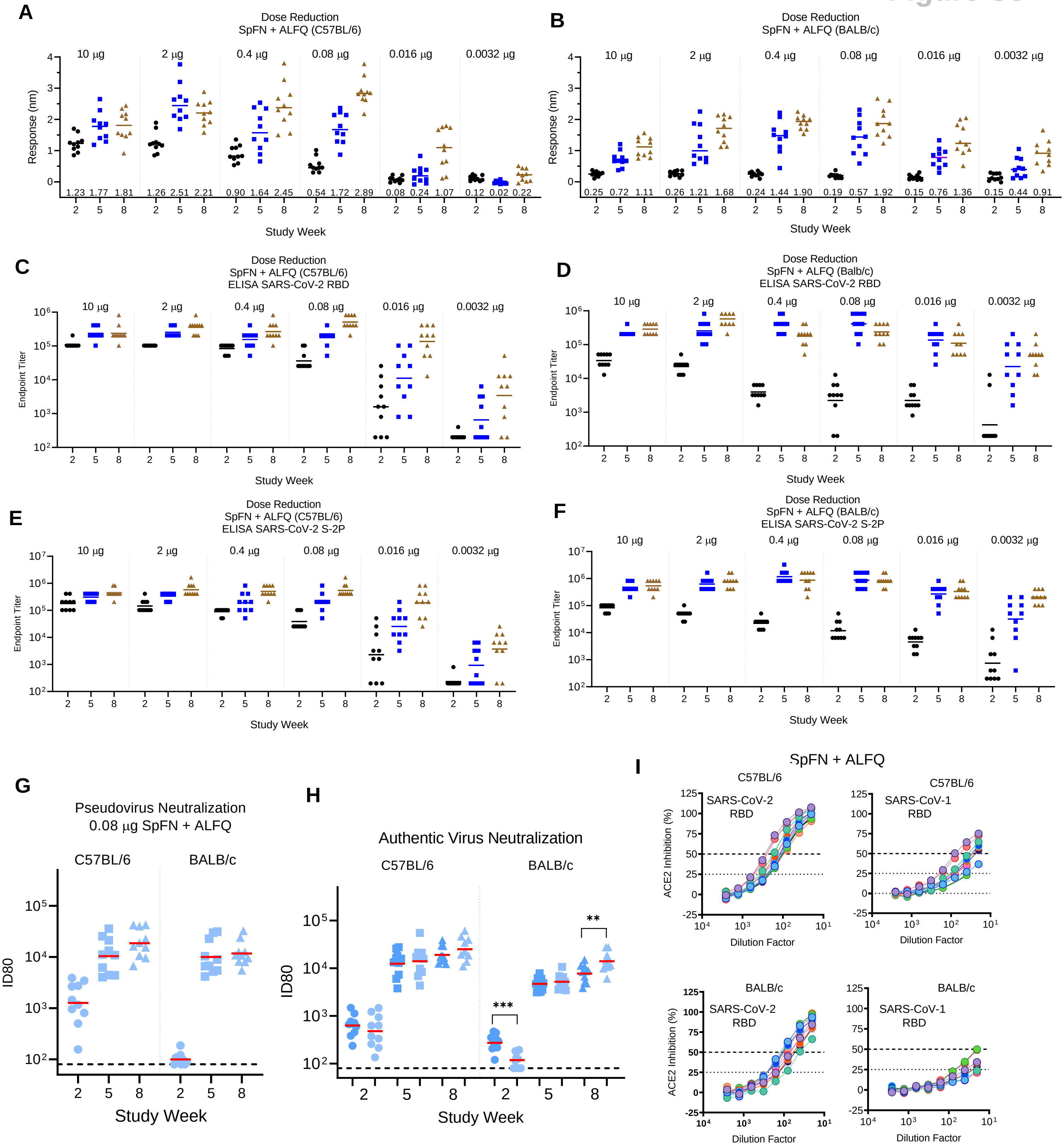

Figure S5 SARS-CoV-2 SpFN vaccine candidate elicits robust binding and neutralizing antibody responses at reduced doses in mice. Related to Figure 5 and 7.
(A) Biolayer interferometry analysis of C57BL/6 and (B) BALB/c mouse sera binding response to SARS-CoV-2 RBD following immunization with reducing doses of SpFN.
(C, E) ELISA analysis of C57BL/6 and (D, F) BALB/c mouse sera binding response to SARS-CoV-2 RBD or S-2P following immunization with reducing doses of SpFN.
(G) SARS-CoV-2 pseudovirus ID80 neutralization titers of mice immunized with 0.08 µg SpFN + ALFQ.
(H) Authentic SARS-CoV-2 virus ID80 neutralization titers of mice immunized with 10 µg (blue) or 0.08 µg (light blue) SpFN + ALFQ. Geometric mean titers for each group and time point are indicated by a horizontal line, n =10. Neutralization titers for the two dose groups at each study time point were compared for statistically significant differences using a Mann-Whitney unpaired two-tailed non-parametric test. The two BALB/c time points that showed differences are indicated by bars. P values <0.001 (***), <0.01 (**).
(I) Mouse sera from study week 10 was analyzed for hACE2 blocking capacity to SARS-CoV-2 RBD (left) or SARS-CoV-1 RBD using a biolayer interferometry assay format.

bioRxiv preprint doi: https://doi.org/10.1101/2021.05.09.443331; this version posted May 10, 2021. The copyright holder for this preprint (which was not certified by peer review) is the author/funder. This article is a US Government work. It is not subject to copyright under 17 USC 105 and is also made available for use under a CC0 license.

Figure S6

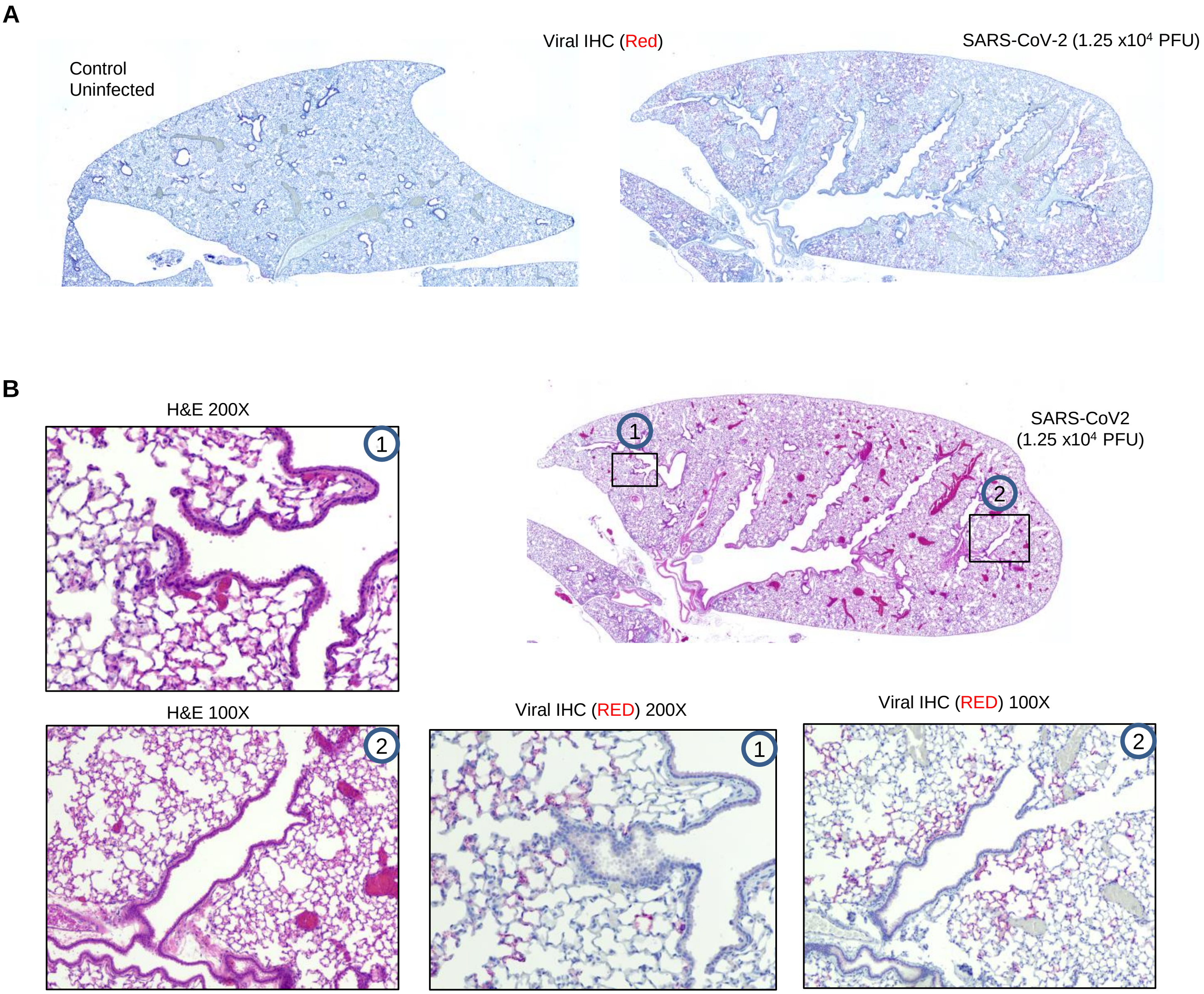

Figure S6 Histopathological analysis of SARS-CoV-2 infection in K18-ACE2 mice. Related to Figure 7
(A, B) Hematoxylin and eosin staining of lung sections from K18-hACE2 mice following intranasal infection with 1.25 x 10^4 PFU SARS-CoV-2. Images show two magnifications. Images are representative of n = 10 per group.
